# Autoantibodies as drivers of neuropathic pain

**DOI:** 10.1016/j.molmed.2025.07.003

**Published:** 2026-02

**Authors:** Adham Farah, Omar Daifallah, Evanka Singh, John M. Dawes

**Affiliations:** 1 Nuffield Department of Clinical Neurosciences, University of Oxford, OX3 9DU, UK; 2 Department of Zoology, King Saud University, Riyadh, Saudi Arabia

## Abstract

Emerging evidence, supported by clinical responses to immunotherapy and the recapitulation of sensory symptoms in passive transfer models, shows that autoantibodies (AAbs) may drive neuropathic pain. These findings highlight the importance of immune profiling to enhance diagnosis and treatment, and provide molecular insights into broader pain mechanisms in clinical contexts.

## Neuropathic pain and AAbs

Neuropathic pain, which affects 7–10% of the population, remains a significant clinical challenge despite the availability of current analgesics, highlighting the urgent need to understand mechanisms and develop more targeted treatment strategies. It has long been recognised that inflammation, and consequently the immune system, can enhance pain sensitivity following tissue injury and these neuroimmune interactions can contribute to neuropathic pain [1]. More recently, autoimmune mechanisms, including AAbs targeting the nervous system, have been implicated in neuropathic pain [1]. For example, AAbs targeting specific antigens have been identified (Table 1) in patients, with pain improved following the use of immunotherapies, suggesting that these AAbs are not merely incidental but pathogenic. Most current evidence arises from well-defined conditions [2], although the overall prevalence of AAb-mediated neuropathic pain is unclear. While some of these conditions are rare, when the target protein is known [e.g., contactin-associated protein 2 (CASPR2) or leucine-rich glioma-inactivated 1 (LGI1)], they can provide unparalleled insights into potentially common molecular pathways sufficient to cause pain in patients. In contrast, AAb mechanisms may contribute more widely, with studies indicating involvement in nerve trauma [3] and AAbs may underlie idiopathic conditions as suggested in small fibre neuropathy (SFN) [4].

**Table 1:** Identified AAbs associated with neuropathic pain in patients

Target	Target details	Pain mechanism	Passive transfer model	Refs
CASPR2	Transmembrane protein that promotes the clustering of potassium channels, contributing to voltage-gated potassium channel (VGKC) complex localization, axon formation, and stability	Sensory neuron hyperexcitability via disrupting Kv1 channels on sensory neurons	Mouse model shows mechanical pain hypersensitivity	[5,7]
LGI1	Secreted protein and key component in the VGKC complex. Plays an important role in the formation and maintenance of synapses	Unclear, but LGI1 known to regulate Kv1 channels and AMPA receptors	Not done	[5,11]
CRMP5	Neuronal protein involved in axon guidance, synaptic signalling, and neuronal differentiation	Increased excitability of sensory neurons	Rat models show mechanical pain hypersensitivity	[10]
Plexin D1	Transmembrane semaphorin receptor that plays a role in cell signalling and involved in axon guidance and neuronal development	Cytoskeletal dynamics ERK and MAPK signalling	Mouse model shows mechanical and thermal pain hypersensitivity	[4]
AQP4	Water channel protein that facilitates the movement of water across cell membranes	Activation of astrocytes and release of ATP	Rat model shows mechanical pain hypersensitivity	[9]

The study of AAbs is facilitated by the availability of patient samples (e.g., blood) allowing researchers unique access to the putative factors that may be driving neuropathic pain and therefore the ability to assess causality and understand the underlying pathogenic mechanisms.

## AAbs are causal to neuropathic pain

The fact that treatments targeting AAbs, such as intravenous immunoglobulin (IVIG) or plasma exchange, relieve pain more effectively than standard analgesics in patients with AAb involvement, provides evidence that these factors play a causal role in pain [5,6].

One approach to more directly assess causality is the use of passive transfer models, where purified patient IgG is introduced into experimental animals. A number of studies have shown that these models recapitulate patient pain characteristics, supporting the idea of patient AAbs being capable of driving neuropathic pain [4,6., 7., 8.]. One of the first examples was the passive transfer of CASPR2 AAbs from neuropathic pain patients into mice which resulted in significant mechanical pain hypersensitivity [7]. This study used systemic delivery over a 2–3-week period to elevate serum levels. This approach more closely resembles the human situation, and assessment of mouse tissue identified the dorsal root ganglia (DRG) but not CNS as the location of action. Other studies have used more local delivery methods. For example, in two separate studies, IgG from SFN patients, either targeting plexin-D1 or derived from patients with Guillain–Barré syndrome (GBS), were delivered intrathecally, to target the DRG leading to increased mechanical and thermal pain-related hypersensitivity [4,6]. Intraspinal delivery has been used to assess the pathogenicity of AAbs targeting aquaporin-4 (AQP4) associated with neuromyelitis optica [9]. In some circumstances, additional factors are required for the pathogenic effects to emerge. For example, complex regional pain syndrome (CRPS) patient IgG does not induce pain in naïve mice but instead exacerbates pain only in injured mice, highlighting the potential need for prior tissue injury supported by recent studies in traumatic nerve injury models [3]. While passive transfer models have been effective in demonstrating the ability of patient AAbs to cause pain, it should be considered that they often rely on high-dose, short-term IgG administration; therefore not replicating the longer-term immune interactions that occur in patients.

Another approach is the use of immunisation models to induce the production of AAbs against a selected target. Paraneoplastic AAbs targeting collapsin response mediator protein 5 (CRMP5) are associated with painful neuropathies in patients and the immunisation of rats to produce their own CRMP5 AAbs results in mechanical pain hypersensitivity which is also observed following the transfer of patient CRMP5 AAbs [10]. In addition to providing evidence for antibody pathogenicity, these models may also offer insights into immunological and cellular mechanisms [9,10]. Other immunoglobulin isotypes, such as IgM and IgA, have been less extensively studied but may also contribute to neuropathic pain.

## How do antibodies cause pain?

**Figure 1:**
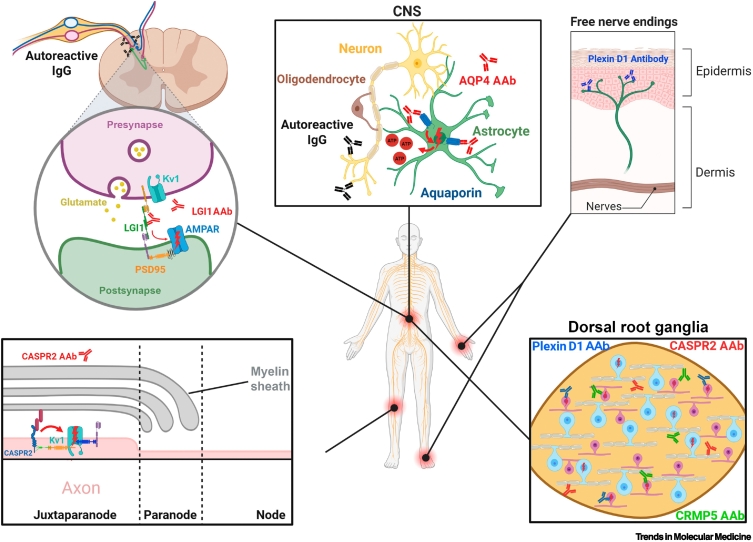
Schematic representation of autoantibody (AAb)-mediated pain mechanisms across the nervous system. Overview (top left): the figure illustrates multiple potential sites of action for pathogenic AAbs, highlighting their roles in altering neuron and glia function across the central and peripheral nervous systems. Red arrows and red lightning bolts represent pathogenic antibody binding and its downstream effects. Spinal cord/CNS: AAbs targeting aquaporin-4 (AQP4) on astrocytes may cause excessive ATP release, and disruption of astrocyte–neuron signalling. AAbs against leucine-rich glioma-inactivated 1 (LGI1) may disrupt the LGI1–ADAM22–AMPAR complex at the post-synapse and Kv1 channels at the presynapse, contributing to central sensitisation and pain. Autoreactive IgG may target cells in the spinal cord (and DRG) following nerve injury axon: AAbs (may target axonal compartments to disrupt ion channel distribution. For example, contactin-associated protein 2 (CASPR2) which is highly concentrated at the juxtaparanode of the node of Ranvier. Dorsal root ganglia (DRG): AAbs against collapsin response mediator protein 5 (CRMP5) (green) and CASPR2 (red) alter ion channel expression and contribute to hyperexcitability of sensory neurons. Plexin D1 (blue) AAbs can bind small-diameter sensory neurons to activate intracellular pathways such as pERK, increasing neuronal sensitivity. Free nerve endings: plexin D1 AAbs, associated with small fibre neuropathy may target intraepidermal nociceptors in the skin. Created with BioRender.com.

The development of animal models, along with the application of patient AAbs to *in vitro* systems has facilitated investigation into the mechanisms by which these antibodies contribute to pain (Figure 1).

### Direct neuronal effects

AAbs can disrupt neuronal physiology by targeting ion channels. Well-studied targets of AAbs include neuronal proteins such as CASPR2 and LGI1, which can form complexes with ion channels such as Kv1.1 and Kv1.2 subunits, important determinants of sensory neuron excitability. Multiple *in vitro* studies on mouse DRG neurons show that patient CASPR2 AAbs directly bind to these neurons and disrupt Kv1 channel expression and function, leading to DRG neuron hyperexcitability; a characteristic feature of neuropathic pain [7]. Similarly, patient CRMP5 AAbs induce hyperexcitability of cultured rat DRG neurons potentially through regulation of ion channels by CRMP5 and its interaction with CRMP2 [10].

Plexin D1 AAbs from SFN patients target nociceptive soma and fibres. Rather than having explicit actions on ion channels, through their binding to this semaphorin receptor, these AAbs activate intracellular pathways (e.g., pERK) which are known to contribute to increased nociceptor activity and enhanced pain sensitivity [4].

### Fcγ receptor and immune-complex mechanisms

A recent study suggests that in some cases peripheral nerve injury itself can trigger pathogenic AAb production, as demonstrated by the increase of IgG in DRG and spinal cord and prevention of neuropathic pain with B cell depletion therapies [3]. These AAbs may not target surface neuronal antigens but instead develop against nerve injury products to form immune complexes that activate Fcγ receptors) on sensory neurons and immune cells, driving neuroinflammation and neuronal hyperexcitability. In line with the involvement of the Fcγ receptor, its genetic removal in mice reduces the overactivity of sensory neurons in response to nerve injury and prevents the development of neuropathic pain behaviours [3].

### Effects on glial cells

AAbs can also affect non-neuronal cells. Astrocytes play a key role in neuropathic pain development and the passive transfer of patient AQP4 AAbs has been shown to target spinal astrocytes. These AAbs induce astrocytes to release excessive levels of ATP (levels in patients are also elevated) increasing ATP signalling in spinal cord. This is associated with enhanced mechanical pain sensitivity which can be relieved with ATP blockers [9]. Furthermore, patient CRPS IgG may also work through non-neuronal mechanisms inducing activation of microglia and astrocytes in the spinal cord [8].

## Therapeutic implication and future directions

AAbs associated with neuropathic pain sometimes have known targets and respond well to immunotherapy, so testing for these antibodies should be considered, especially in idiopathic cases. In other conditions, such as CRPS, pathogenic AAbs may contribute to pain, but their precise targets remain unknown. Identifying these targets would improve diagnosis and treatment. In some circumstances, AAbs can directly impact neuronal physiology through target disruption, potentially revealing proteins relevant to human pain. For example, the genetic ablation of CASPR2 or LGI1 (targets of AAbs in neuropathic pain patients) increases pain sensitivity in mice [7,11], identifying novel pain pathways with therapeutic potential.

Effective immunotherapies already exist for AAb-mediated pain conditions. Treatments such as IVIG and plasma exchange can relieve pain in autoimmune neuropathies, including chronic inflammatory demyelinating polyneuropathy (CIDP), SFN, and certain seropositive cases such as those with CASPR2 or LGI1 AAbs [5,12., 13., 14.]. These therapies reduce antibody levels and, in many cases, lead to clinical improvement where standard analgesics have failed. Although responses to these therapies can vary, they provide a proof-of-concept that targeting immune mechanisms is a viable treatment strategy. New approaches are now emerging, including anti-CD20 therapies (e.g., rituximab), which deplete B cells and neonatal Fc receptor blockers that prevent the recycling of pathogenic AAbs; both of which reverse pain in animal models [10,15].

These advances also underscore the importance of identifying the right patients for immunotherapy. While known AAbs can directly guide treatment, in other cases immune-mediated pain may still occur in the absence of a defined target. This highlights the need for broader or novel diagnostic approaches. For example, current assays could be applied more widely in patients with clinical features suggestive of immune involvement, such as rapid symptom onset, poor response to conventional therapies, or comorbid autoimmune diseases. Additionally, platforms that screen patient antibodies against primary neurons could help to uncover new antigens and the identification of those patients most likely to benefit from immunotherapy.

All these developments point to a future in which stratification of neuropathic pain based on immune profiling will allow for more personalized and effective treatments.

## Concluding remarks

Growing evidence strongly supports the idea that AAbs that target antigens within the nervous system can drive neuropathic pain in patients. Looking ahead, it is essential to improve identification of patients whose neuropathic pain is mediated by AAbs, allowing for more targeted diagnosis and intervention. This will require wider adoption of immune profiling techniques in clinical research and neuropathic pain cohorts to fully characterise the prevalence and clinical significance of these mechanisms. Furthermore, results from the study of AAbs provides insights into clinically relevant pain mechanisms which may be applicable to the understanding and potential treatment of neuropathic pain more generally.
